# The long non-coding RNA LIMT inhibits metastasis of hepatocellular carcinoma and is suppressed by EGF signaling

**DOI:** 10.1007/s11033-022-07325-0

**Published:** 2022-05-08

**Authors:** Yu Hu, Hao Li, Hongwei Zhang, Qiang Tang, Guangtan Zhang, Xiqing Li, Fei Xue

**Affiliations:** 1 Department of Hepatobiliary and Pancreatic Surgery, Henan Provincial People’s Hospital, Henan University People’s Hospital, 450003 Zhengzhou, Henan Province PR China; 2 Department of Hepatobiliary and Pancreatic Surgery, Henan Provincial People’s Hospital, Zhengzhou University People’s Hospital, Henan University People’s Hospital, 450003 Zhengzhou, Henan Province PR China; 3 Department of Gastrointestinal Surgery, Henan Provincial People’s Hospital, Zhengzhou University People’s Hospital, Henan University People’s Hospital, 450003 Zhengzhou, Henan Province PR China; 4 Department of Oncology, Henan Provincial People’s Hospital, Zhengzhou University People’s Hospital, Henan University People’s Hospital, 450003 Zhengzhou, Henan Province PR China

**Keywords:** LIMT, Epithelial-mesenchymal transition, EGF, Hepatocellular carcinoma

## Abstract

**Background:**

The long non-coding RNA LIMT (lncRNA inhibiting metastasis) acts as a tumor suppressor factor in some cancers. However, the biological role of LIMT in hepatocellular carcinoma (HCC) has not been explored.

**Methods and Results:**

Quantitative real-time PCR was performed to evaluate the expression of LIMT in HCC tissue. The effects of LIMT on tumor growth and metastasis were assessed by *in vitro* experiments, including colony formation and transwell assays, and *in vivo* in nude mouse models. Western blot analysis was used to evaluate the expression levels of proteins associated with epithelial-mesenchymal transition (EMT). LIMT expression was significantly lower in HCC than in normal liver tissue. Functionally, overexpression of LIMT repressed the proliferation, invasion, and EMT of HCC cells, while LIMT knockdown increased proliferation, invasion, and EMT of HCC cells *in vitro*. Furthermore, LIMT overexpression suppressed HCC growth and metastasis while silencing of LIMT had an opposite effect *in vivo*. Finally, LIMT overexpression reversed EGF-induced EMT.

**Conclusions:**

Our results suggest that LIMT could play an anti-cancer effect in HCC and might be a potential novel therapeutic target in HCC.

**Supplementary Information:**

The online version contains supplementary material available at 10.1007/s11033-022-07325-0.

## Introduction

Hepatocellular carcinoma (HCC) is one of the most prevalent cancers, and is associated with a high mortality rate [1]. Despite great progress in earlier diagnosis and targeted treatment of HCC, the prognosis for patients with HCC remains unsatisfactory, due in large part to high recurrence and metastasis rates after surgery [2]. Therefore, in order to improve clinical outcomes in HCC, it is necessary to identify novel diagnostic and therapeutic targets and to further elucidate mechanisms of HCC recurrence and metastasis.

Long noncoding RNAs (lncRNAs) are a type of RNA greater than 200 nucleotides in length that are not translated into protein, but that have significant functional characteristics, including involvement in the pathology of many diseases and in the progression of malignant tumors [3–5]. Dysregulation of lncRNAs is closely associated with tumor progression, during which lncRNAs act as critical mediators through transcriptional and post-transcriptional regulation [6, 7]. For example, lncRNA SNHG11 is highly expressed in HCC tumor tissues and cells and functions as an oncogene [8]. LncRNA MAFG-AS1 promotes malignancy in ovarian cancer by upregulating NFKB1-dependent IGF1 [9]. Conversely, lncRNA KAT7 is significantly downregulated in colorectal cancer (CRC) tissues, and overexpression of KAT7 can inhibit malignant behavior in CRC [10]. Additionally, overexpression of lncRNA GAS5 impairs the proliferation and migration of bladder cancer cells, and also promoted apoptosis in bladder cancer cells [11]. LncRNAs also can serve as independent risk factors in breast cancer diagnosis [12], and have prognostic value for other malignant tumors [13].

Recently, some it has been demonstrated that the lncRNA LIMT (lncRNA inhibiting metastasis, also called LINC01089) inhibits tumor growth in some cancers, including gastric cancer, colorectal cancer, and breast cancer [14–16]. Notably, Chen et al. first reported that LIMT is downregulated in breast cancer and suppressed by EGF [17]. At the same time, the low expression of LIMT predicts poor prognosis in breast cancer. However, the role of LIMT in HCC has not been investigated.

In this study, we compared the expression of LIMT in tumor tissue and normal tissue, and we found that LIMT was down-regulated in HCC tissue. *In vitro* and *in vivo* experiments were performed to investigate the biological functions of LIMT in HCC. Next, the relation between LIMT and EGF was explored. Our data reveal novel functions for LIMT in HCC, and suggest that LIMT may be a potential novel target for HCC treatment.

## Materials and methods

### Collection of HCC samples

After obtaining informed consent from patients, 45 pairs of HCC tissues and normal tissue were collected, immediately frozen in liquid nitrogen, and stored at − 80 °C. All experiments conducted in this study were approved by the Ethics Committee of Henan Provincial People’s Hospital.

### Cell lines and transfection

Human HCC cell lines, Huh-7 and LM3, were purchased from the Cell Bank of Chinese Academy of Sciences (Shanghai, China). Cell lines were cultured in DMEM supplemented with 10% fetal bovine serum (FBS) and kept in a humidified atmosphere at 37 °C with 5% CO_2_.

The pcDNA empty vector (Vector), pcDNA-LINC01089 overexpression vector (LINC01089), siRNA negative control (si-NC), and siRNAs against LINC01089 (si-LINC01089) were provided by GenePharma (Shanghai, China). Cell transfection was carried out according to the manufacturer’s instructions. After 6 h post-transfection, cells were cultured in fresh complete medium for subsequent experiments.

### Cell invasion assays

Transwell assays were performed to evaluate cell invasion by using transwell chambers with 8-µm pore size inserts (NEST, Jiangsu, China). Briefly, the upper chamber was coated with Matrigel (1:8 diluent). Next, 200 µL cell suspension (about 1 × 10 ^5^ cells in serum-free media) were added into the upper chamber, and 700 µL media containing 10% FBS was added into the lower chamber. After 24 h of routine culture, the Matrigel gel and any cells remaining on the upper surface of the membrane were gently wiped off. Cells that invaded through the membrane were fixed with 4% paraformaldehyde for 30 min, and stained with 1% crystal violet for 3 min. Cells were observed and counted under an inverted microscope.

### Real-time quantitative reverse-transcription polymerase chain reaction (qRT-PCR)

RNAiso Plus (Takara, Dalian, China) was used to isolate total RNA from HCC tissues and cell lines, according to the manufacturer’s protocol. Total RNA was reverse transcribed into cDNA using PrimeScript™RT Master Mix form RNA (Takara). The cDNA was then subjected to RT-PCR on an ABI 7500 Fast Real-Time PCR System with TB Green® Premix Ex Taq™ (Takara). The primers used in this study are listed in the supplementary file. The expression levels of LIMT were quantified using 2^−ΔΔCT^ method.

### Western blot analysis

Total protein extraction from cells and subsequent western blot determination of protein levels was carried out according to our previous methods [18]. The primary antibodies used in this study are listed in the supplementary file.

### Colony formation assay

Transfected cells were lifted with 0.25% trypsin, re-suspended, and counted. Next, 400 cells/well were seeded into 6-well plates containing 2 ml culture medium and incubated in a 5% CO_2_ humidified incubator at 37 °C for 2 weeks. The cells were washed with PBS, fixed with 10% formalin, stained with 0.1% crystal violet and counted.

### *In vivo* assays

For *in vivo* experiments, 6-week-old male BALB/c-nu nude mice were purchased from Nanjing GemPharmatech Co., Ltd. For the metastasis assay, 32 mice were randomly divided into normal saline, sh-NC, shRNA-LIMT, vector and exp-LIMT groups (n = 8). Briefly, 1.0 × 10^6^ LM3 cells were suspended in 25 µl physiological saline solution and injected into the liver envelope of the left outer lobe of mice. After 4 weeks, mice were injected intraperitoneally with D-luciferin (75 mg/kg), and photographed within 30 min. For the tumor growth assay, vector-LM3/exp-LIMT-LM3 cells were subcutaneously injected into the armpit of the nude mice. Tumor formation was observed every 2 days and tumor size was measured. Fourteen days after tumor cell injection, mice were euthanatized by overdose of anesthesia, and the tumors were removed for follow-up evaluation. All animal studies were approved by the Animal Care Ethics Committee of Henan Provincial People’s Hospital and performed in accordance with the institutional guidelines.

### Statistical analysis

All experiments were performed in at least three independent replicates and data were expressed as the means ± standard deviation (SD). For comparisons, Student’s t test was used to evaluate the differences between two groups. Statistical analysis was performed with GraphPad Prism 7.0 (San Diego, CA, USA). A p-value of P < 0.05 was considered to indicate a statistically significant difference.

## Results

### LIMT expression is decreased in HCC tissue

**Fig. 1:**
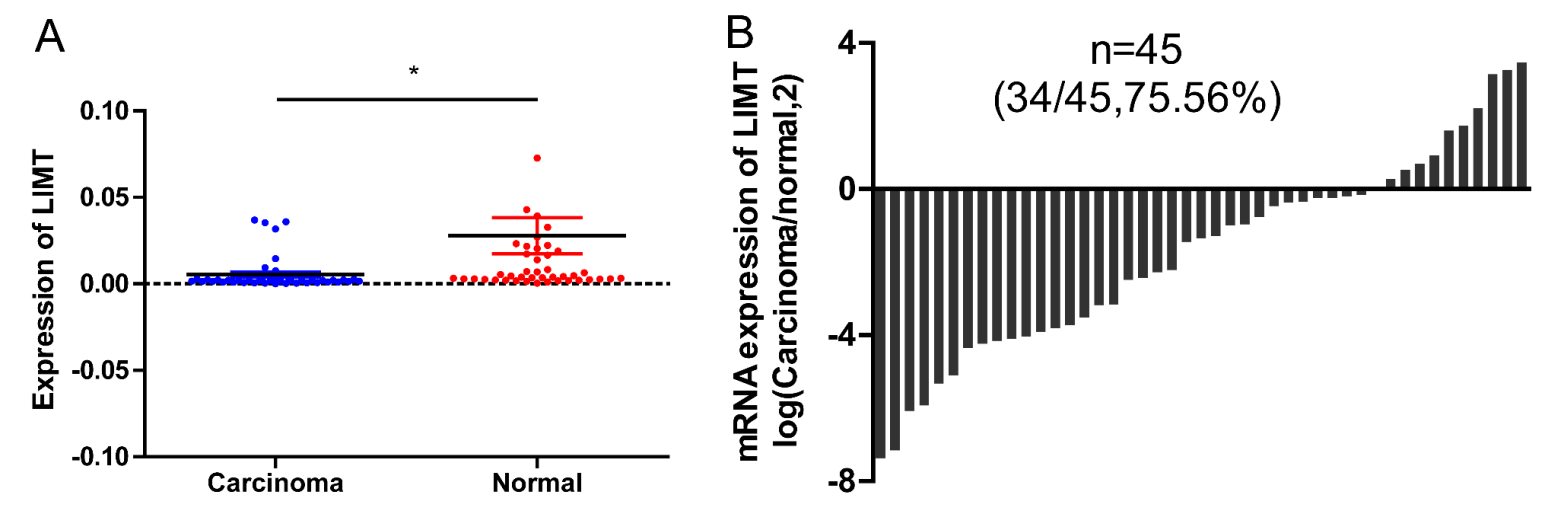
**The expression of LIMT is lower in HCC samples than in normal tissue** (A) LIMT expression was evaluated in 45 paired HCC samples and normal samples using RT-qPCR. *p < 0.05. (B) Comparison of LIMT mRNA levels in HCC tissues and normal tissues

Initially, we used RT-qPCR to evaluate the endogenous expression of LIMT in 45 pairs of HCC tissues and normal tissues. We found that LIMT was significantly underexpressed in HCC tissue compared with paired normal tissue (Fig. 1 A). Among the 45 sample pairs, 34 pairs showed low expression of LIMT in the HCC sample, implying that the aberrant expression of LIMT might participate in HCC progression (Fig. 1B).

### LIMT attenuates invasion and clonogenic ability of HCC cells and inhibits the epithelial-mesenchymal transition (EMT)

**Fig. 2:**
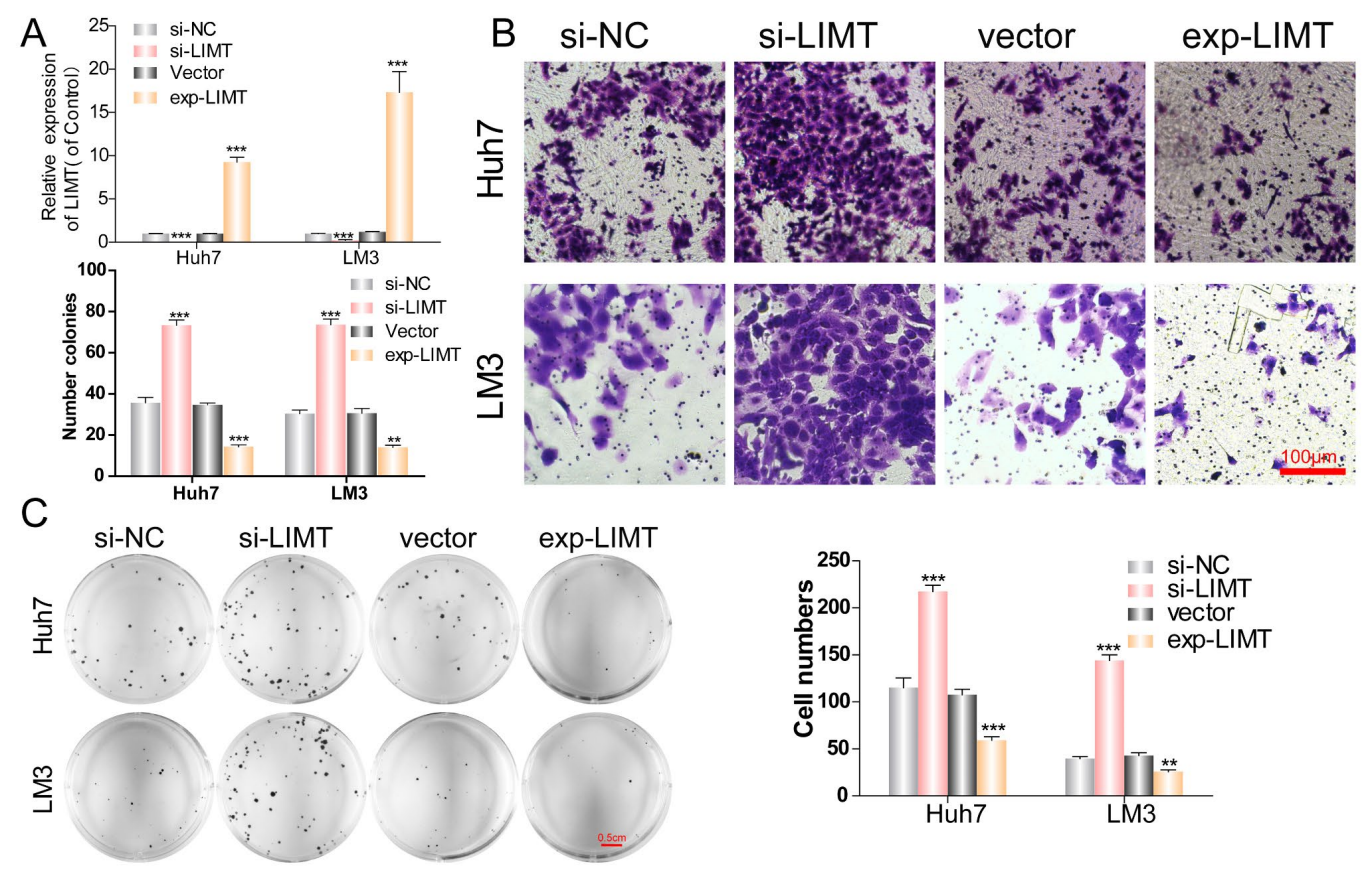
**LIMT attenuates the invasion and clonogenic ability of HCC cells** (A) Huh7 and LM3 cells were transfected with the LIMT overexpression plasmid or siRNAs targeting LIMT, respectively, and the transfection efficiency was investigated by qRT-PCR. ***p < 0.001. (B) Transwell assays were employed to investigate the invasion of Huh7 and LM3 cells after transfection. ***p < 0.001. (C) Cell colony formation assays were used to evaluate the clonogenic ability of Huh7 and LM3 cells after transfection. **p < 0.01,***p < 0.001

Next, we investigated the biological function of LIMT on HCC cells. In Huh-7 and LM3 cells, knockdown of LIMT expression was accomplished using LIMT-targeting siRNA, and an overexpression plasmid was used to overexpress LIMT. The transfection efficiency was measured by qRT-PCR (Fig. 2 A). According to the transwell assay, knockdown of LIMT significantly increased the number of migrated cells in Huh-7 and LM3 cells. In contrast, LIMT overexpression repressed the invasion of Huh-7 and LM3 cells, as evidence by a reduced number of migrated cells (Fig. 2B). Depletion of LIMT promoted the proliferation of Huh-7 and LM3 cells in the colony formation assay, while LIMT overexpression inhibited colony formation in both cell lines (Fig. 2 C).

**Fig. 3:**
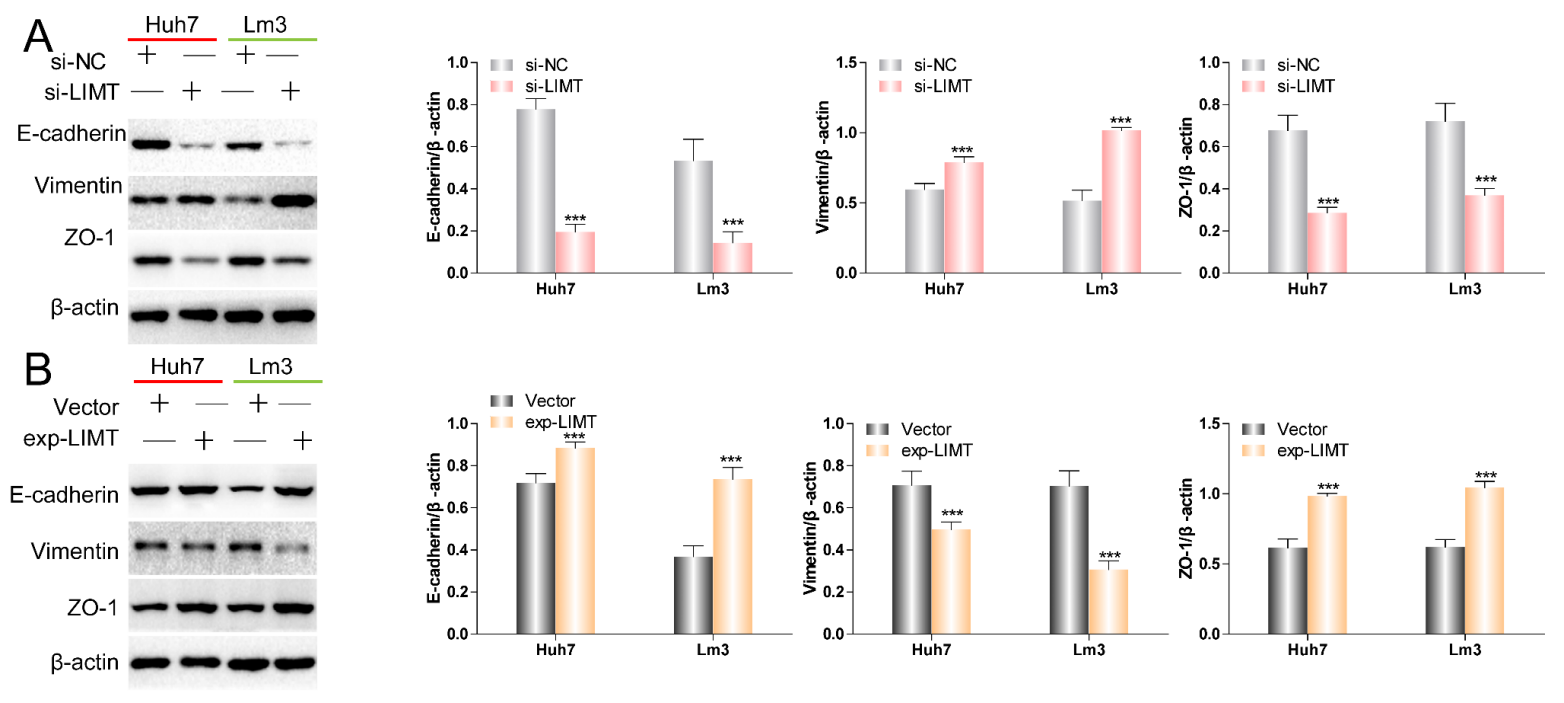
**LIMT affects the expression of EMT-related markers** (A-B) Western blot was utilized to examine the expression of EMT markers in Huh7 and LM3 cells after transfection. ***p < 0.001

Tumor metastasis involves several processes, including EMT, cell migration, resistance to anoikis, and angiogenesis [19]. EMT, as the first step, is involved in tumor occurrence and progression [20]. Therefore, we also investigated the relationship of LIMT expression and EMT. Following knockdown of LIMT, expression of E-cadherin and ZO-1 was decreased, while Vimentin expression was increased (Fig. 3 A). Conversely, upregulation of LIMT resulted in marked increases in E-cadherin and ZO-1 expression and reduction of Vimentin expression (Fig. 3B). These data indicate that LIMT might exert an antitumor effect in HCC by mediating EMT.

### LIMT inhibits tumor growth and metastasis *in vivo*

**Fig. 4:**
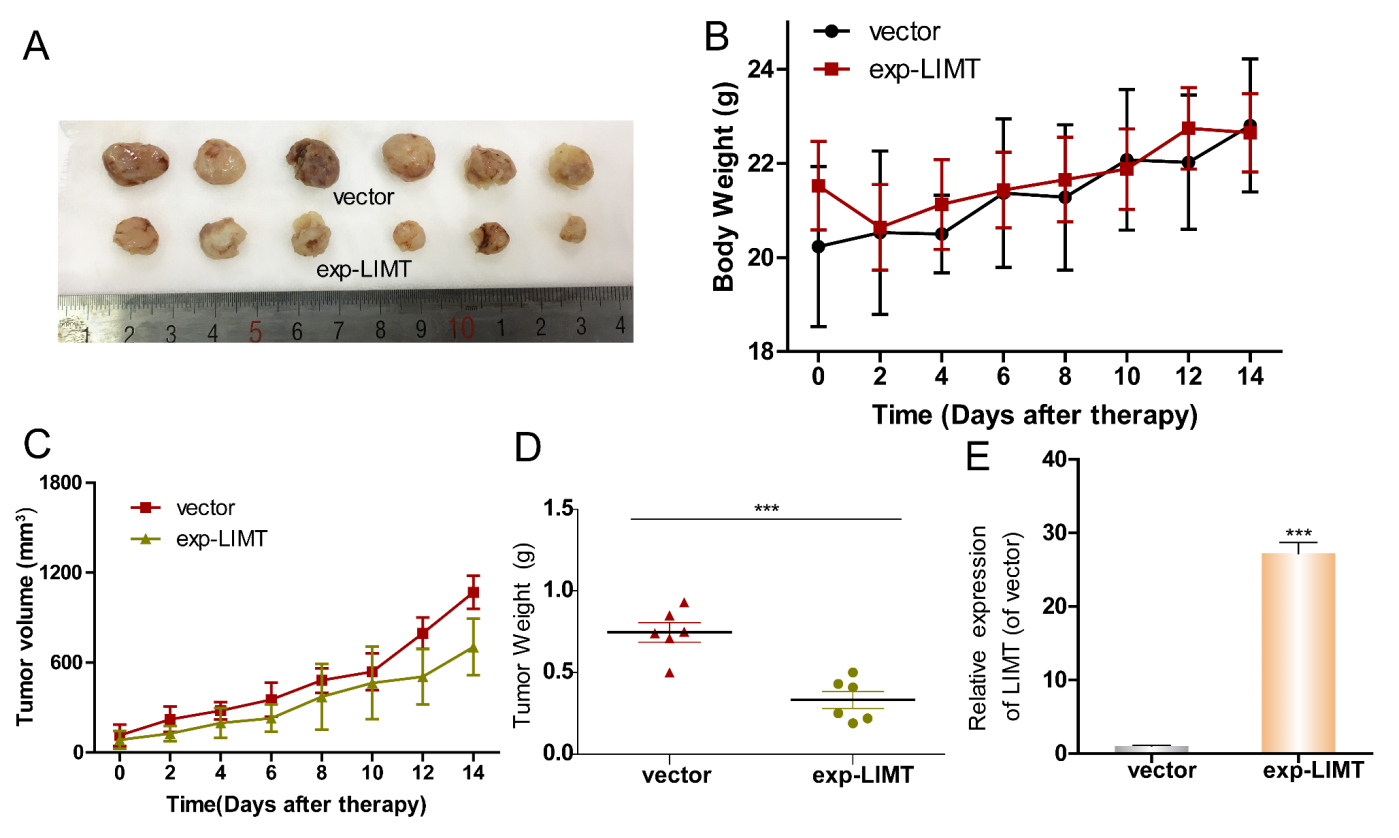
**LIMT inhibited tumor growth** ***in vivo***. (A) Representative images of tumors. (B) Body weight and (C) tumor growth curves for groups treated with or without exp-LIMT. (D) Tumor weight for groups treated with or without exp-LIMT. (E) LIMT expression level in tumors. ***P < 0.001

To further explore the function of LIMT on tumor growth *in vivo*, vector-LM3/exp-LIMT-LM3 cells were subcutaneously injected into the armpit of nude mice. Compared with vector group, the tumor sizes of mice treated with exp-LIMT were significantly reduced (Fig. 4 A). There was no difference in body weight between the two groups (Fig. 4B). The tumor volumes and weights of the exp-LIMT group were significantly lower than the vector-LM3 groups (Fig. 4 C-D). Before injection, LIMT expression in vector-LM3/exp-LIMT-LM3 cells was assessed by RT-PCR (Fig. 4E).

**Fig. 5:**
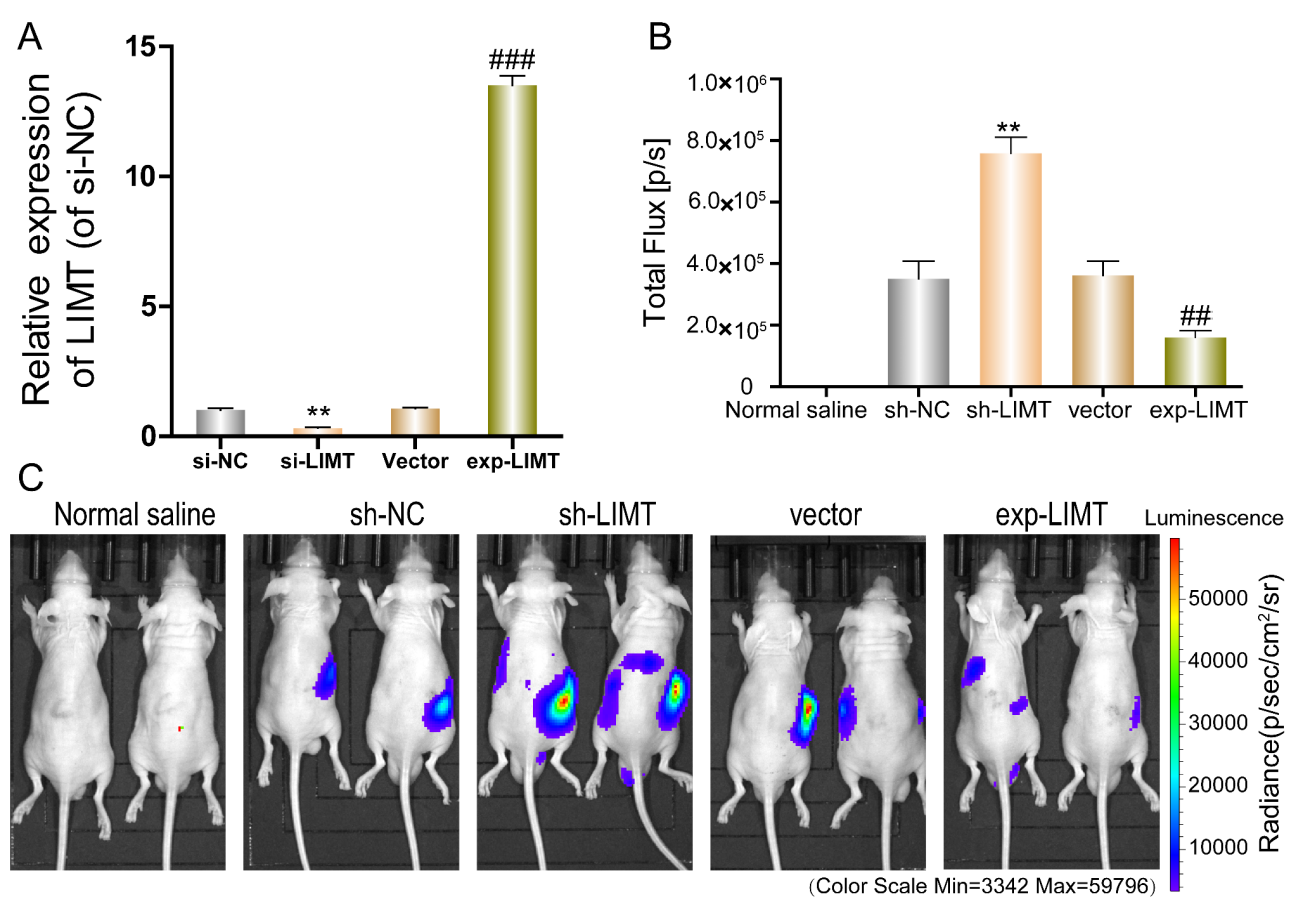
**The effects of LIMT on metastasis** ***in vivo*** (A) The transfection efficiency was investigated by qRT-PCR before injection. **p < 0.01 vs. si-NC. ### p < 0.001 vs. vector (B-C) A total of 1 × 10^6^ transfected LM3 cells were injected into the liver envelope of the left outer lobe of mice. Tumors were monitored every two days using an *in vivo* imaging system. The imaging and quantification of mean fluorescence intensity are shown. **p < 0.01 vs. si-NC. ## p < 0.01vs vector

To illustrate the effects of LIMT on HCC metastasis, we built an *in vivo* model of LIMT ectopic expression by transfecting LIMT overexpression plasmid or LIMT shRNAs into LM3 cells. Before injection, the transfection efficiency was measured by qRT-PCR (Fig. 5 A). By comparing the fluorescence intensity of the five groups, we found that fluorescence was not expressed in the normal saline group and there was no difference in fluorescence levels between the sh-NC group and vector group. Fluorescence intensity in the sh-LIMT group was dramatically stronger than in the sh-NC group, and was lower in the LIMT overexpression group compared with the vector group (Fig. 5B-C). We also observed tumor metastasis in the sh-LIMT group, suggesting that LIMT expression may be associated with tumor metastasis (Fig. 5 C). Altogether, these results indicate that LIMT could suppress tumor growth and metastasis during HCC progression.

### LIMT reversed EGF-induced invasion and EMT *in vitro*

**Fig. 6:**
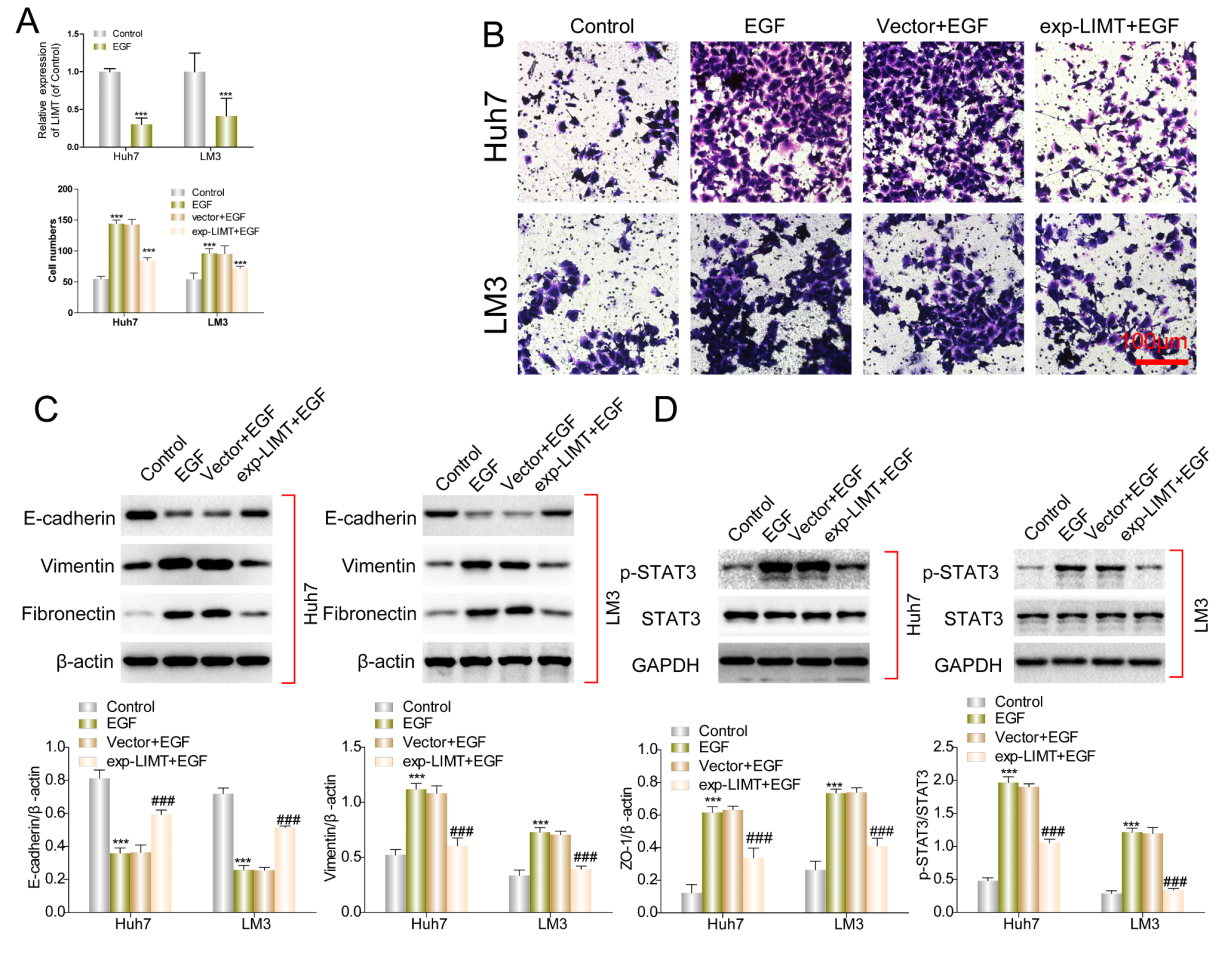
**LIMT reversed the effect of EGF on HCC cells** (A) The expression level of LIMT was examined by qRT-PCR in HCC cells following EGF treatment. ***p < 0.001 (B) Transwell assays were used to evaluate potential of LIMT-overexpressing HCC cells stimulated with EGF. ***p < 0.001 (C-D) Western blot assays were used to evaluate the expression of a series of proteins in LINC01089-overexpressing HCC cells stimulated with EGF. ***p < 0.001 vs. control, ###p < 0.001 vs. vector + EGF group

Given that LIMT is suppressed by EGF [17], and EGF induces EMT [21], we explored the relationship of LIMT and EGF-induced EMT in HCC. After EGF stimulation, LIMT expression was lower in both HCC cell lines than in cells untreated with EGF (Fig. 6 A). We found that EGF significantly stimulated cell invasion in both Huh7 and LM3 cells, whereas exp-LIMT inhibited the EGF-induced invasion (Fig. 6B). Meanwhile, EGF significantly induced EMT in HCC cells, as indicated by a decrease in protein levels of E-cadherin and an increase in protein levels of Vimentin and Fibronectin in both Huh7 and LM3 cells (Fig. 6 C). LIMT overexpression partially reversed the EMT induced by EGF (Fig. 6 C). The transcription factor STAT3 is a downstream target of the EGFR pathway [22], and we found that EGF stimulation activated expression of p-STAT3, which was reversed by exp-LIMT (Fig. 6D). Besides, according to Starbase 3.0 analysis, we found that LIMT expression was negatively correlated with STAT3 expression (Fig. S1). Taken together, these data demonstrate that LIMT inhibited EGF-induced EMT *in vitro*.

## Discussion

In the past 10 years, lncRNA has become a topic of intense interest in cancer research. LncRNAs play important roles in HCC by regulating cell proliferation, migration, and invasion [23–25]. The lncRNA LIMT has been identified as a novel and critical regulator in breast cancer [17]. However, the role of LIMT in HCC has not been investigated. In the current study, we found that LIMT was underexpressed in HCC tissues, compared to matched normal tissue. Loss- and gain-of-function experiments demonstrated that LIMT overexpression restrained the proliferation and invasion of HCC, while LIMT under-expression promoted the proliferation and invasion of HCC cells. Moreover, *in vivo* experiments demonstrated that LIMT inhibits tumor growth and metastasis. These data suggested that LIMT plays an inhibitory role in HCC, which is consistent with previous reports [26].

Metastasis is the main cause of cancer-related death [27], and is characterized by a multi-step process that allows cancer cells to spread from the primary tumor and colonize in distant organs by obtaining molecular and phenotypic changes. EMT is a necessary initial step for tumor metastasis. Many lncRNAs are reported to be involved in tumor metastasis by modifying EMT [28, 29]. LIMT is a class of lncRNAs that are associated with inhibiting metastasis [17]. We found that LIMT inhibited HCC metastasis by inhibiting EMT. Knockdown of LIMT led to a decrease of E-cadherin and ZO-1 expression, and an increase of Vimentin expression. Conversely, LIMT overexpression increased E-cadherin and ZO-1 expression, and reduced Vimentin expression. Many EMT-induced transcription factors, including Snail, Slug, and twist, are implicated in tumor invasion and metastasis [21]. However, the specific factors that are regulated by LIMT to mediate tumor invasion and metastasis require further elucidation.

Regulation mechanisms of lncRNA can be very complex. Nuclear lncRNAs mainly regulate gene expression at the transcriptional level, while cytoplasmic lncRNAs generally regulate gene expression at the post-transcriptional level through competing endogenous RNA (‘ceRNA’); indeed, the same lncRNA can have different functions in the nucleus and in the cytoplasm [30]. We previously studied the role of miRNAs in HCC [31, 32]. Therefore, we hypothesized that LIMT may have anti-tumor effects through adsorption of miRNA. In the future, we will further explore the function of nuclear LIMT as a potential ceRNA in HCC.

It has been demonstrated that LIMT functions as a tumor-suppressor lncRNA that can be regulated by EGF [17, 33]. EGF, like other tyrosine kinase receptor ligands (TGF-b, FGF, IGF), can induce EMT [21].Thus we want to know whether LIMT is regulated by EGF and the role of LIMT in EGF-induced EMT in HCC. In this work, we observed that LIMT expression decreased after EGF treatment. EGF enhanced cell proliferation and invasion and induced EMT, which was partially reversed by upregulation of LIMT; this was consistent with a previous report [33]. EGF induces EMT in cancers through the EGF/EGFR signaling pathway, thus promoting the invasion and metastasis of tumor cells [34, 35]. Similarly, we also found that the activation of EGF/EGFR signal pathway was partially inhibited by LIMT overexpression, as evidenced by the reduction of p-STAT3. These data demonstrate that LIMT is regulated by EGF and LIMT can inhibit EGF-induced invasion and EMT.

In conclusion, we demonstrate here that LIMT was underexpressed in HCC samples. *In vitro* experiments showed that LIMT markedly suppressed proliferation and invasion of HCC cells mainly by inhibiting EMT. Our *in vivo* studies demonstrated that LIMT suppressed HCC tumor growth and metastasis. Additionally, we showed that LIMT expression was regulated by EGF. These results highlight the potential role of LIMT in regulating HCC metastasis.

## Electronic Supplementary Material

Below is the link to the electronic supplementary material.

Supplementary Material 1

## Data Availability

The data used to support the findings of this study are included within the article.
