# Supplementary material for: The long non-coding RNA LIMT inhibits metastasis of hepatocellular carcinoma and is suppressed by EGF signaling
Source: Mol Biol Rep. 2022 May 8;49(6):4749–57. doi: 10.1007/s11033-022-07325-0 (PMC9262785; doi:10.1007/s11033-022-07325-0)
Supplement: Supplementary file 1 — Supplementary Material 1 [file 11033_2022_7325_MOESM1_ESM.docx]

Table 1. Primer sequence

| Gene | Sequence |
| --- | --- |
| LIMT  GAPDH | F: 5’-CGAATGGACAATCTTTCCTTCTGTC  R:3’-GCTAGAGGTTGAGGGCCTGAGT  F:5’- TGTTGCCATCAATGACCCCTT  R:3’- CTCCACGACGTACTCAGCG |

Table 2. siRNA sequence

| Gene | Sense(5’-3’) | Antisense(3’-5’) |
| --- | --- | --- |
| LINC01089-homo-177  LINC01089-homo-295  LINC01089-homo-888  NC | CCAUUCAUGUCAGCAGUUATT  GCAGAACGUGAGGGUGUAATT  GCUUCCAACCUCCAUUGCATT  UUCUCCGAACGUGUCACGUdTdT | UAACUGCUGACAUGAAUGGTT  UUACACCCUCACGUUCUGCTT  UGCAAUGGAGGUUGGAAGCTT  ACGUGACACGUUCGGAGAAdTdT |

Table 3 the information of antibodies used.

| Antibody name | company | Cat. # |
| --- | --- | --- |
| E-Cadherin  Vimentin  ZO-1  Fibronectin  β-actin  GAPDH | CST  CST  CST  Proteintech  CST  CST | 3195s  3390S  8193T  66042  4970  2118S |

Supplementary figure 1


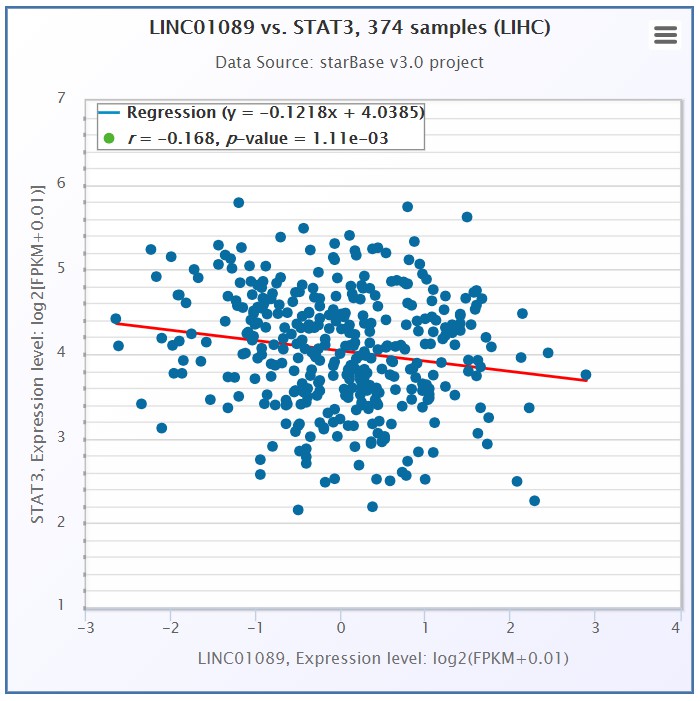


Figure S1 LIMT is negative associated with STAT3.
